# Hepatitis E Virus Infection among Solid Organ Transplant Recipients, the Netherlands

**DOI:** 10.3201/eid1805.111712

**Published:** 2012-05

**Authors:** Suzan D. Pas, Rob A. de Man, Claudia Mulders, Aggie H.M.M. Balk, Peter T.W. van Hal, Willem Weimar, Marion P.G. Koopmans, Albert D.M.E. Osterhaus, Annemiek A. van der Eijk

**Affiliations:** Erasmus Medical Center, Rotterdam, the Netherlands

**Keywords:** hepatitis E, hepatitis E virus, viruses, solid organ transplant, the Netherlands, lung transplant, kidney transplant, heart transplant, liver transplant, infection

## Abstract

We screened 1,200 living heart, lung, liver, and kidney transplant recipients for hepatitis E virus infection by reverse transcription PCR. In 12 (1%) patients, hepatitis E virus infection was identified; in 11 patients, chronic infection developed. This immunocompromised population is at risk for hepatitis E virus infection.

Hepatitis E virus (HEV) can cause acute or chronic infection in humans. Four genotypes have been identified in humans. HEV genotype 3 predominantly infects pigs and deer, but is also recognized as a zoonotic agent. As awareness increases, more reports of HEV infection among humans, especially immunocompromised persons, have been published (*1*,*2*).

Analysis of exposure histories of persons with HEV genotype 3 infections has demonstrated its underdiagnosis, and a source was not identified for most cases (*3*). Because HEV has been reported as a cause of liver disease in solid organ transplant (SOT) recipients (*4*), we screened all living recipients of SOTs during 2000–2011 at Erasmus Medical Center, the largest SOT center in the Netherlands, for HEV RNA. This study was designed to identify SOT recipients with acute or chronic HEV infection.

## The Study

A cross-sectional study was performed of all living adult SOT recipients for whom serum or EDTA-plasma samples were available in the Erasmus Medical Center biobank (stored at –20°C and –80°C, respectively, and collected during previous routine visits to the outpatient clinic; complete methods are described in detail in the Technical Appendix). Some recipients eventually had been referred to peripheral hospitals. A Laboratory Information Management System database search was performed for availability of the most recent follow-up sample. Thirty-nine HEV RNA–positive samples in the center’s biobank from non-SOT patients were genotyped and used as reference for phylogenetic analysis. Samples were screened for HEV RNA by using real-time reverse transcription PCR (RT-PCR) (*5*) with primers detecting all 4 genotypes and validated according to International Standards Organization guidelines 9001 and 15189 (www.iso.org/iso/search.htm). HEV IgM and IgG were detected by using the PE2 HEV-IgM and IgG ELISA (Wantai Biological Pharmacy Enterprise Co., Ltd., Beijing, People’s Republic of China). A case of HEV infection was defined by the following criteria: an HEV RNA–positive sample, confirmed either by presence of HEV IgM or IgG or HEV RNA in sequential samples. Chronic infection was diagnosed by retrospective testing of stored samples and defined as HEV RNA positive for >6 months. We retrospectively tested samples from HEV RNA–positive patients so the antibody kinetics and viremia levels could be studied. For calculating phylogenetic relationships, HEV open reading frame (ORF) 1 sequences were generated with primer set MJ-C (*6*). All viral sequences were deposited into GenBank (accession nos. JQ015399–JQ015448).

The 1,200 SOT recipients consisted of 259 heart transplant (HTX), 53 lung transplant (lungTX), 300 liver transplant (LTX), 574 kidney transplant (NTX), and 14 multiple SOT recipients (4 HTX–NTX, 1 lungTX–NTX, and 9 LTX–NTX). Twelve HEV-infected patients were identified: 5 HTX, 1 lungTX, 3 LTX, and 1 NTX recipients and 2 multiple SOT-recipients (1 HTX–NTX and 1 LTX–NTX). For 11 patients, HEV infection was chronic (Table 1). The median age of the HEV-infected patients was 56.9 years (range 19.9–63.5 years); 9 (75%) were men. In 10 HEV patients, immunosuppression was achieved by using prednisolone and tacrolimus, combined with mycophenolate mofetil (n = 3) or everolimus (n = 2). Two patients received regimens of cyclosporine and prednisolone or mycophenolate mofetil and prednisolone.

**Table 1:** Overview of HEV infections among SOT recipients, the Netherlands, 2000–2011*

SOT group	No. recipients	HEV infections, no. (%)	
		Confirmed	Chronic
HTX	259	5 (1.9)	5 (1.9)
LungTX	53	1 (1.9)	1 (1.9)
LTX	300	3 (1.0)	3 (1.0)
NTX	574	1 (0.2)	1 (0.2)
Multiple SOT†	14	2 (14.3)	1 (7.1)
Total	1,200	12 (1.0)	11 (0.9)

*HEV, hepatitis E virus; SOT, solid organ transplant; HTX, heart transplant; lungTX, lung transplant; LTX, liver transplant; NTX, kidney transplant. †9 NTX–LTX, 4 NTX–HTX, and 1 NTX–lungTx.

All patients who had chronic HEV infection had elevated liver enzyme levels; bilirubin levels were elevated in 45.5% of the patients (Table 2). Although it proved difficult to identify abnormal liver functions uniquely related to the HEV infection, HEV RNA detection always coincided with or was followed by an increase in alanine aminotransferase. Apparently no overt clinical symptoms were associated with infection; however, such symptoms are difficult to recognize in immunosuppressed SOT recipients. Inflammation compatible with viral hepatitis was shown in 8 of 9 patients with chronic infection for whom liver biopsy specimens were available. Other findings were F0–F2 fibrosis, steatosis 1–2 (Brunt classification), cholestasis, and Councilman bodies.

**Table 2:** Parameters in chronic HEV infections among SOT recipients, the Netherlands, 2000–2011*

Parameter	Median	Range	ULN (F/M)
Peak alanine aminotransferase, U/L	301	81–909	30/40
Peak aspartate aminotransferase, U/L	172	66–1016	30/36
Peak gamma-glutamil transferase, U/L	299	72–1740	34/49
Peak bilirubin, μmol/L	16	5–100	16/16
Peak HEV RNA, cycle threshold values	20.0	16.7–26.6	NA
Period of HEV RNA positivity, mo	16	6–55	NA
Time between SOT and first HEV RNA–positive result, mo	2.0	–0.3 to 20.1	NA
Time of HEV RNA positivity before HEV IgM positive, d	32	0–826	NA
Time of HEV RNA positivity before HEV IgG positive, d	124	0–826	NA

*HEV, hepatitis E virus; SOT, solid organ transplant; ULN (F/M), upper limit of normal (female/male); NA, not applicable.

Samples from all 12 HEV patients were tested for HEV RNA and HEV IgM and IgG. One infection was traced to 2003 (lungTX), 1 to 2008 (NTX), 1 to 2009 (multiple SOT recipient, NTX–HTX), 7 to 2010 (5 HTX, 1 LTX and 1 multiple SOT recipient, NTX–LTX) and 3 to 2011 (all LTX). Among the patients, 1 LTX recipient had an acute HEV infection and cleared the virus within 6 days. Because HEV IgM and IgG were detected 4 years before HEV RNA detection, both reactivation and reinfection should be considered. The median span of HEV RNA-positive time period of chronic HEV cases was 16 months (range 6–55) with a median peak cycle threshold value of 20.0 (range 16.7–26.6). HEV RNA was detected during viremia (median cycle threshold value 19.9, range 15.5–28.3) in feces from 8 patients with chronic illness.

To assess the value of diagnostic techniques for detection of HEV infection in SOT recipients, we studied antibody kinetics (HEV IgM and IgG) and viremia. The median time from RNA positivity to IgM detection was 32 days (range 0–826 days). Five patients had detectable HEV IgM at the time of HEV RNA positivity. In 1 case, no HEV IgM was detected. HEV IgG titers were detectable an average of 124 days later than HEV RNA (range 0–826 days). HEV IgG was absent in 2 samples, and in 4 samples, HEV IgG was detectable when HEV RNA was detected. The median time between transplantation and first HEV RNA-positive result was –0.3 to 20.0 years (median 1.99 years).

Viruses isolated from samples from 11 HEV-infected patients were all within the genotype 3 group. Because no ORF1b sequences from the Netherlands were available in GenBank, ORF1b sequences were determined from samples from non-SOT HEV-infected patients in the Netherlands (Figure). No indications for a common or nosocomial source of HEV transmission were found.

**Figure:**
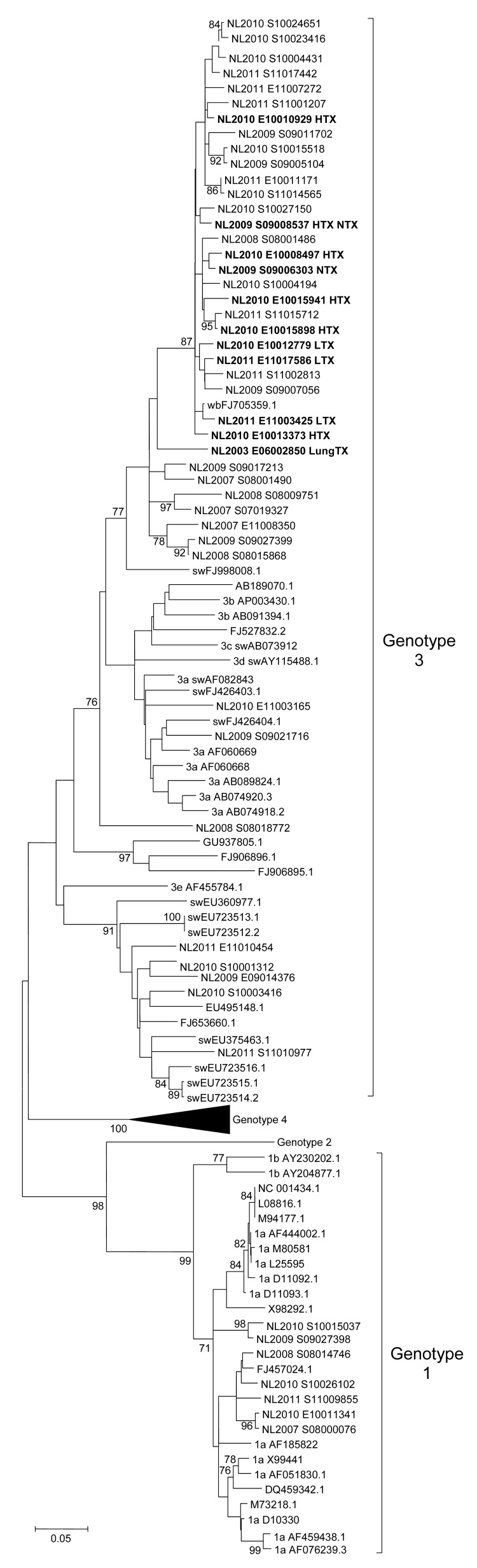
Phylogenetic tree of hepatitis E virus (HEV) open reading frame (ORF) 1 sequences, including HEV infections, the Netherlands, 2000–2011. Phylogenetic relation of a 306-bp ORF1 region was calculated by using maximum-likelihood, Kimura 2-parameter analysis with bootstrapping (n = 1,000). HEV sequences originating in the Netherlands are indicated as NL with year of isolation and isolate number (GenBank accession nos. JQ015399–JQ015448). **Boldface** indicates virus strains of chronic HEV-infected solid organ transplant recipients identified in this study. Scale bar indicates number of nucleotide substitutions per site. HTX, heart transplant; NTX, kidney transplant; LTX, liver transplant; lungTX, lung transplant.

## Conclusions

Recent HEV infections in SOT recipients (*4*,*7*–*9*) prompted us to perform a survey among SOT recipients admitted to the largest transplantation center in the Netherlands. Our findings showed that they are at risk for HEV infection. Nine of 12 case-patients were treated postoperatively with a tacrolimus-based regimen, which has been associated with increased risk for HEV infection (*9*).

The cross-sectional RT-PCR screening detected 12 HEV infections but could not provide information about previously acquired and cleared HEV infections. Real-time RT-PCR screening was performed for 2 reasons. First, because a patient received immunosuppressive drugs, specific antibodies against HEV might be absent. Second, ELISAs have been developed to detect antibodies to genotypes 1 (Myanmar) and 2 (Mexico) and might not be sensitive enough to detect antibodies to genotype 3 or 4 (*10*). Information about results of serologic assays to validate HEV genotype 3 is limited, and seroprevalence measured can vary with the assays used (*11*–*13*). Furthermore, independent studies found that sensitivity and specificity of HEV RNA assays from laboratories in the Netherlands (S.D. Pas and B. Hogema, unpub. data) and other European countries (*14*) differ greatly. Therefore, international standardization should be encouraged.

Although the observed 1% of HEV-infected SOT recipients may seem low, HEV infection may be life threatening in immunocompromised patients. Misdiagnosis of HEV infection as drug-induced liver injury or auto-immune hepatitis has been reported (*15*); empirical treatment of these misdiagnoses by raising immune suppression would exacerbate the condition. Temporary reduction of immunosuppression resulted in immune-mediated control and clearance of HEV in 30% of cases (*9*).

This study also found that in patients with chronic HEV infection, HEV RNA was detected an average of 32–124 days before HEV IgM and IgG, respectively. Therefore, in SOT recipients with elevated liver enzymes (alanine aminotransferase), the diagnosis of HEV infection should be considered and verified by detection of HEV RNA.

This systematic survey of HEV infections among SOT recipients in a major transplant center shows that this population is at risk for HEV infection. Given the consequences of HEV infection, SOT recipients with liver function impairment of unknown etiology should be tested for HEV RNA.

## Supplementary Material

Technical Appendix Complete methods used for cross-sectional study of all living adult solid organ transplant recipients for whom serum or EDTA-plasma samples were available in the Erasmus Medical Center biobank, the Netherlands, 2000–2011.
