# Supplementary material for: Hepatitis E Virus Infection among Solid Organ Transplant Recipients, the Netherlands
Source: Emerg Infect Dis. 2012 May;18(5):869–72. doi: 10.3201/eid1805.111712 (PMC3358074; doi:10.3201/eid1805.111712)
Supplement: Technical Appendix — Complete methods used for cross-sectional study of all living adult solid organ transplant recipients for whom serum or EDTA-plasma samples were available in the Erasmus Medical Center biobank, the Netherlands, 2000–2011. [file 11-1712-Techapp_3p.pdf]

Article DOI: http://dx.doi.org/10.3201/eid1805.111712

Hepatitis E Virus Infection among Solid Organ Transplant Recipients, the Netherlands

Technical Appendix

Materials and Methods

HEV-specific antibody detection

For detection of both HEV specific IgM and specific IgG in serum or plasma samples the commercially available PE2 HEV ELISA (Wantai, Singapore) was used according to the manufacturer's instructions. Samples of HEV-RNA positive patients were retrospectively tested during the entire course of infection to study the kinetics of antibody responses (IgM and IgG) and viremia in the confirmed cases.

HEV-RNA detection

All samples were screened for the presence of HEV RNA by a real-time RT-PCR based on Ahn et al. (*1*) and Zhao et al. (*2*) amplifying a conserved ORF3 region of 77bp. Primers were adapted to detect all four genotypes. Briefly, RNA was extracted using MagnaPureLC (Roche Diagnostics, Almere, The Netherlands) and total nucleic acid isolation kit with an input and output volume of 200µl and 100µl, respectively. The extraction was internally controlled by the addition of a known concentration of Phocine Distemper Virus. Twenty µl extracted RNA was amplified in a 50µl final volume one-step RT-PCR, containing 12.5 µl 4x TaqMan® Fast Virus 1-Step Master Mix (Lifetechnologies, Nieuwerkerk a/d Ijssel, The Netherlands), 0.5 ul (1U/ul) Uracil-N-Glycosylase (Lifetechnologies, Nieuwerkerk a/d IJssel, The Netherlands), 30 pmol HEV fwd (5′-CGGTGGTTTCTGGGGTGA-3′) 45 pmol HEV rev (5′-GCRAAGGGRTTGGTTGG-3′), 5 pmol HEV probe (5′-FAM-ATTCTCAGCCCTTCGC-MGB-3′). The internal control was amplified in a separate reaction using TaqMan Fast Virus 1-Step Master Mix, 0.5 ul (1U/ul) Uracil-N-Glycosylase and primers/probe as described before (*3*). Amplification was performed in a LC480 (Roche Applied Science, Almere, The Netherlands)

using Fit point analysis module. Quality assurance was performed using the free MedlabQC software. Criterion for a successful RT-PCR run was Ct values of both internal control and positive PCR control should be within 3xSD of mean.

Sequence analysis

For phylogenetic analyses ORF1 RdRp (nt 4254–4560) sequences of 306 bp were generated using previously described primer set MJ-C (*4*). Briefly, cDNA was prepared in 50µl volume reaction containing 23µl HEV RNA, 20 pmol EAP 4576–4595 5′-AGGGTGCCGGGCTCGCCGGA-3′, 1x first strand buffer, 0.1M DTT, 10mM dNTP, 80U RNAsin (Promega, Leiden, The Netherlands), 200 units Superscript III RT (LifeTech, Bleiswijk, The Netherlands) and 5 min 65°C and 1 hour 50°C as thermal profile. Subsequently, an outer PCR reaction was performed in a 9700 PCR machine (Applied Biosystems, Nieuwerkerk a/d IJssel, The Netherlands) amplifying 10µl cDNA in a 50µl final PCR mix volume, containing 1x PCR buffer, 5 mM $MgCl_2$, 10 mM dNTP, 2.5 units HotStarTaq DNA Polymerase (Qiagen, Venlo, The Netherlands), 20 pmol ISP 4232–4253 5′-GTATTTCGGCCTGGAGTAAGAC-3′ and 20 pmol EAP 4576–4595 5′-AGGGTGCCGGGCTCGCCGGA-3′. Thermal profile of PCR was 15 min 95^{0}C, 40 cycles of 20″94^{0}C, 30″60^{0}C, 45″72^{0}C and 10′ 72^{0}C. If necessary, a nested PCR was performed using 2 µl outer PCR product, the same reaction conditions and PCR profile as for outer PCR, but ISP 4232–4253 5′-GTATTTCGGCCTGGAGTAAGAC-3′ and IAP 4561–4583 5′-TCACCGGAGTGYTTCTTCCAGAA-3′ as primers. The amplicon was sequenced with 5 pmol of the above-mentioned primers. One microliter of the amplicon was sequenced with the BigDye Terminator version 3.1 cycle sequencing kit (Applied Biosystems, Nieuwerkerk a/d IJssel, The Netherlands). The PCR products were purified using Performa DTR V3 purification plate (Edgebio, Sopachem, Ochten, The Netherlands) and separated on an ABI 3130XL sequencer (Applied Biosystems). The sequence data were analyzed using a Sequence Navigator software sequencer (Applied Biosystems) and SeqMan v9.0 (DNASTAR, Madison,WI). Obtained HEV sequences and GenBank reference sequences (*4*) were aligned and phylogenetic relationships were calculated using maximum likelihood method, Kimura-2 parameter (Mega5.05) and bootstrap resampling (n = 1,000).

References

1. Ahn JM, Rayamajhi N, Gyun Kang S, Sang Yoo H. Comparison of real-time reverse transcriptase-polymerase chain reaction and nested or commercial reverse transcriptase-polymerase chain reaction for the detection of hepatitis E virus particle in human serum. Diagn Microbiol Infect Dis. 2006;56:269–74. PubMed http://dx.doi.org/10.1016/j.diagmicrobio.2006.04.010

2. Zhao C, Li Z, Yan B, Harrison TJ, Guo X, Zhang F, et al. Comparison of real-time fluorescent RT-PCR and conventional RT-PCR for the detection of hepatitis E virus genotypes prevalent in China. J Med Virol. 2007;79:1966–73. PubMed http://dx.doi.org/10.1002/jmv.21040

3. van Doornum GJ, Schutten M, Voermans J, Guldemeester GJ, Niesters HG. Development and implementation of real-time nucleic acid amplification for the detection of enterovirus infections in comparison to rapid culture of various clinical specimens. J Med Virol. 2007;79:1868–76. PubMed http://dx.doi.org/10.1002/jmv.21031

4. Zhai L, Dai X, Meng J. Hepatitis E virus genotyping based on full-length genome and partial genomic regions. Virus Res. 2006;120:57–69. PubMed http://dx.doi.org/10.1016/j.virusres.2006.01.013
